# Effect of Sox10 on remyelination of the hippocampus in cuprizone‐induced demyelinated mice

**DOI:** 10.1002/brb3.1623

**Published:** 2020-05-03

**Authors:** Yu Shao, Juan Ding, Qian‐xiong He, Quan‐rui Ma, Qiang Liu, Chun Zhang, Hao‐wen Lv, Juan Liu

**Affiliations:** ^1^ School of Basic Medical Sciences Ningxia Medical University Yinchuan China; ^2^ Ningxia Key Laboratory of Cerebrocranial Diseases Ningxia Medical University Yinchuan China

**Keywords:** demyelination, hippocampus, remyelination, Sox10

## Abstract

**Objective:**

The low number of oligodendrocytes (OLs) in the hippocampus of patients with schizophrenia suggests that hippocampal demyelination is changed in this condition. Sox10 is expressed throughout OL development. The effect of Sox10 on myelin regeneration is unknown. This study aimed to analyze changes in Sox10 expression in the hippocampus and its regulatory role in hippocampal myelin regeneration in a mouse model of demyelination.

**Methods:**

Mice were fed 0.2% cuprizone (CPZ) for six weeks to establish the acute demyelinating model (CPZ mice). Behavioral changes of these mice were assessed via open field and tail suspension tests. The ultrastructure of the myelin sheaths in the hippocampus was observed by transmission electron microscopy. The expression levels of myelin sheath‐related proteins and the transcription factor Sox10 were detected via immunohistochemistry and Western blots. Furthermore, Sox10‐overexpressing adeno‐associated virus was injected into the hippocampus after establishing the demyelinating model to investigate effects of Sox10 on remyelination.

**Results:**

CPZ mice showed abnormal behavioral changes, a large number of pathological changes in the myelin sheaths, and significantly reduced protein expression of the myelin sheath markers myelin basic protein and proteolipid protein. This confirmed that the demyelinating model was successfully established. Meanwhile, the protein expression of the oligodendrocyte precursor cell marker neural/glial antigen 2 (NG2) increased, whereas Sox10 expression decreased. After Sox10 overexpression in the hippocampus, the abnormal behavior was improved, the ultrastructure of the myelin sheaths was restored, and the expression of myelin sheath protein was reversed. NG2 expression was upregulated.

**Conclusion:**

Overexpression of Sox10 promotes hippocampal remyelination after CPZ‐induced acute demyelination.

## Graphical abstract

To investigate the effect of Sox10 on remyelination of hippocampus in CPZ‐induced demyelinating mice. Overexpression of Sox10 promotes hippocampal remyelination after CPZ‐induced acute demyelination.

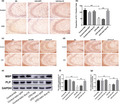

## 1 INTRODUCTION

Schizophrenia is a common mental illness with unknown etiology. Over the last decade, an increasing number of studies have confirmed that neuropathological changes due to myelin sheath injury contribute to schizophrenia (Zhang et al., 2018). Clinical evidence suggests that abnormal hippocampal structure and function are closely related to schizophrenia (Papiol et al., 2017). Compared with healthy individuals, patients with schizophrenia feature smaller hippocampal volumes but no loss of neurons. Hippocampal network function is disrupted in schizophrenia and these disruptions are associated with relational memory ability, suggesting that resting hippocampal network modularity may be an important marker of neuropathology in schizophrenia (Avery, Rogers, & Heckers, 2018). Interestingly, the expression of myelin basic protein (MBP) is reduced in the hippocampus of a demyelination animal model of schizophrenia (Cumberland, Palliser, Rani, Walker, & Hirst, 2017). A quantitative stereological study further confirmed that the number of oligodendrocytes (OLs) is decreased in the hippocampus of patients with schizophrenia (Schmitt et al., 2009). This result suggests that hippocampal demyelination occurs in schizophrenia. Genomic studies have shown that OLs and myelin‐related genes in the hippocampus of patients with schizophrenia are abnormally expressed (Katsel, Davis, & Haroutunian, 2005). Therefore, the change in hippocampal demyelination may be a pathological basis of schizophrenia. OLs are myelin‐forming cells in the central nervous system (CNS). Apoptosis or death of OLs leads to the demyelination of a large number of nerve fibers, resulting in the massive loss of axons and nerve cells and blocked or disordered signal transduction (Takahashi, Sakurai, Davis, & Buxbaum, 2011). But the precise mechanism remains unclear. The research had found that sex‐determining region Y‐related HMG box 10 (Sox10) is a transcription factor that is expressed throughout the development of OLs. Its main role is to influence the terminal differentiation of oligodendrocyte precursor cells (OPCs) and myelination. However, the effects of Sox10 on myelin regeneration are unknown. Cuprizone (CPZ) can induce demyelination in the CNS and has been widely used for studying the myelin abnormality hypothesis of schizophrenia (Benardais et al., 2013). Therefore, we used CPZ to establish an acute demyelinating mouse model and observed the changes of Sox10 expression in the hippocampus and its regulatory effect on hippocampal remyelination. It may provide a new therapeutic target for the treatment of myelin sheath injury in schizophrenia.

## 2 MATERIALS AND METHODS

### 2.1 Animals

Six weeks old healthy ICR male mice (18–22 g) were provided by the Experimental Animal Center of Ningxia Medical University. The mice were allowed to drink water and eat ad libitum at control ambient temperature (22°C). All experiments were carried out in accordance with the National Institutes of Health Guide for Care and were approved by the Experimental Animal Ethics Committee of Ningxia Medical University (Ethical No. 2014–014).

### 2.2 Preparation of the demyelinating model

The demyelinating model was prepared by feeding mice with 0.2% CPZ (Sigma‐Aldrich) for six weeks (Wang et al., 2020). The control group was fed a normal diet.

### 2.3 Stereotactic injection

Mice were randomly divided into six groups (*n* = 15/group). Targeted injection into the hippocampus was performed with normal saline (NS), adeno‐associated virus‐green fluorescent protein (AAV‐GFP), and Sox10 overexpressing adeno‐associated virus (AAV‐Sox10). The groups of mice and treatments were as follows: (a) Control + NS, (b) Control + AAV‐GFP, (c) Control + AAV‐Sox10, (d) CPZ + NS, (e) CPZ + AAV‐GFP, and (f) CPZ + AAV‐Sox10. Mice were anesthetized intraperitoneally with chloral hydrate (4%, 0.01 ml/g) before the intracranial stereotaxic injection of AAV or NS through the mouse stereotaxic apparatus (RWD LIFE SCIENCE). Details of the stereotaxic injection were described previously (Keiser, Chen, & Davidson, 2018). Briefly, we made a longitudinal incision to expose the bregma, set this point as zero, and determined the hippocampus region from this point (AP ± 2 mm, ML ± 2 mm, DV ± 2 mm). Each side of the hippocampus was injected with 1 μl for five minutes. Then, the needle was retained for five minutes before it was slowly retracted. The same method was performed on the other side. One to two needles were used to stitch the wound. The mice were sacrificed after being fed with normal food for two weeks.

### 2.4 Open field test

The open field test (OFT) was designed to observe the autonomous and exploratory behavior of experimental animals in a new environment. Activity time around the corner and center time indicates avoidance and anxiety levels, respectively (Fischer et al., 2015). The box was 30 cm high and 50 cm long at the bottom. A digital camera was set at the top. Mice were placed in the open field laboratory in advance to allow adaptation to the environment for two hours. Before the test began, the box was swabbed with 75% ethanol and dried with a dry cloth. The mice (*n* = 15/group) were placed with fixed orientation toward one side of the box wall and left to explore the environment freely for five minutes. We recorded the time in the central area, time in the corner area, time in the side area, and the number of defecations (Liu et al., 2019). After each mouse experiment ended, the experimenters cleaned the defecation and swabbed the open box again to prevent the smell of one mouse affecting the next test.

### 2.5 Tail suspension test

The tail suspension test (TST) was designed to assess the level of depression in mice (Jiang et al., 2018). The tail of mice (*n* = 15/group) was clamped and suspended so that the mice were hanging upside down while their head was about 10 cm away from the bottom, facing the camera. The experiment took six minutes, and the camera system recorded the immobility of the mice lasting four minutes (Chatterjee, Jaiswal, & Palit, 2012). The immobility time is the time when the mice gave up their struggle and expressed disappointment. This time reflects the emotional changes of the mice.

### 2.6 Transmission electron microscopy

The mice (*n* = 3/group) were anesthetized by intraperitoneal injection of 4% chloral hydrate at 0.01 ml/g and then perfused with 4% paraformaldehyde. The hippocampus was exfoliated and immersed in 2% glutaraldehyde for two hours, followed by 0.1 M sodium dimethyl arsenate solution for two hours (three times), soaked in 1% osmium tetroxide for two hours, and sodium dimethyl arsenate solution for 15 min (two times). The tissues were dehydrated in ethanol and propylene oxide series. Afterward, the specimen was immersed in complete embedding solution at 35°C for six hours, placed at 42°C overnight, and then at 60°C for 48 hr (Kim, Sohn, Kim, & Kim, 2018). After solidification and sectioning, the ultrastructure of hippocampal myelin was observed via transmission electron microscopy (TEM).

### 2.7 Immunohistochemistry

Paraffin sections (*n* = 6/group) were successively placed into xylene, high‐to‐low concentration alcohol, and then repaired antigen. Sections were incubated with goat serum for 15 min. Mouse monoclonal antibody Sox10 (1:500, Cat# ab212843, Abcam), rabbit polyclonal antibody neural/glial antigen 2 (NG2; 1:500, Cat# ab129051, Abcam), rabbit polyclonal antibody proteolipid protein (PLP; 1:500, Cat# ab28486, Abcam), mouse monoclonal antibody MBP (1:500, Cat# ab62631 Abcam), and mouse monoclonal antibody CC‐1 (1:500, Cat# OP80, Merck Millipore) as the primary antibodies were added to the sections and incubated at 37°C for 60 min. Secondary antibodies were applied at room temperature for 20 min. DAB was applied for 5–10 min (Wang et al., 2018). Hematoxylin staining solution was applied for 20 s. The sections were dehydrated and transparently sealed with neutral gum. Images were acquired under an optical microscope (Olympus BX51).

### 2.8 Western blot

Total protein was extracted from hippocampal tissues (*n* = 6/group), and the concentration of the protein samples was detected via the BCA protein assay kit (KeyGEN BioTECH). Proteins were separated using SDS‐PAGE and then electroblotted onto PVDF membranes. The membranes were incubated with 5% skimmed milk for two hours. Anti‐mouse monoclonal antibody Sox10 (1:1,000), anti‐rabbit polyclonal antibody NG2 (1:1,000), anti‐rabbit polyclonal antibody PLP (1:1,000), and anti‐mouse monoclonal antibody MBP (1:1,000) as the primary antibodies were added to the membrane at room temperature for one hour and then overnight at 4°C. Then, the membranes were incubated with the secondary antibodies at room temperature for one hour. The bands were visualized using the enhanced chemiluminescence method. The band intensity was analyzed using Image J software.

### 2.9 Statistical analysis

SPSS18.0 software was used for statistical analysis of the data. Measurement data with normal distribution assessed via a normality test were expressed as mean ± standard error of the mean (*SEM*). Abnormally distributed data were described as median ± quartile distance. For comparison of multiple groups of data, ANOVA was used for differential analysis if the data were normal and the variance was homogeneous. The Bonferroni test was used for comparison between the two groups. The nonindependent sample Kruskal–Wallis test was used if the variances were not equal. Statistical significance was considered at *p* < .05.

## 3 RESULTS

### 3.1 Effect of CPZ on the behavior of mice and the ultrastructure of hippocampal myelination

**Figure 1:**
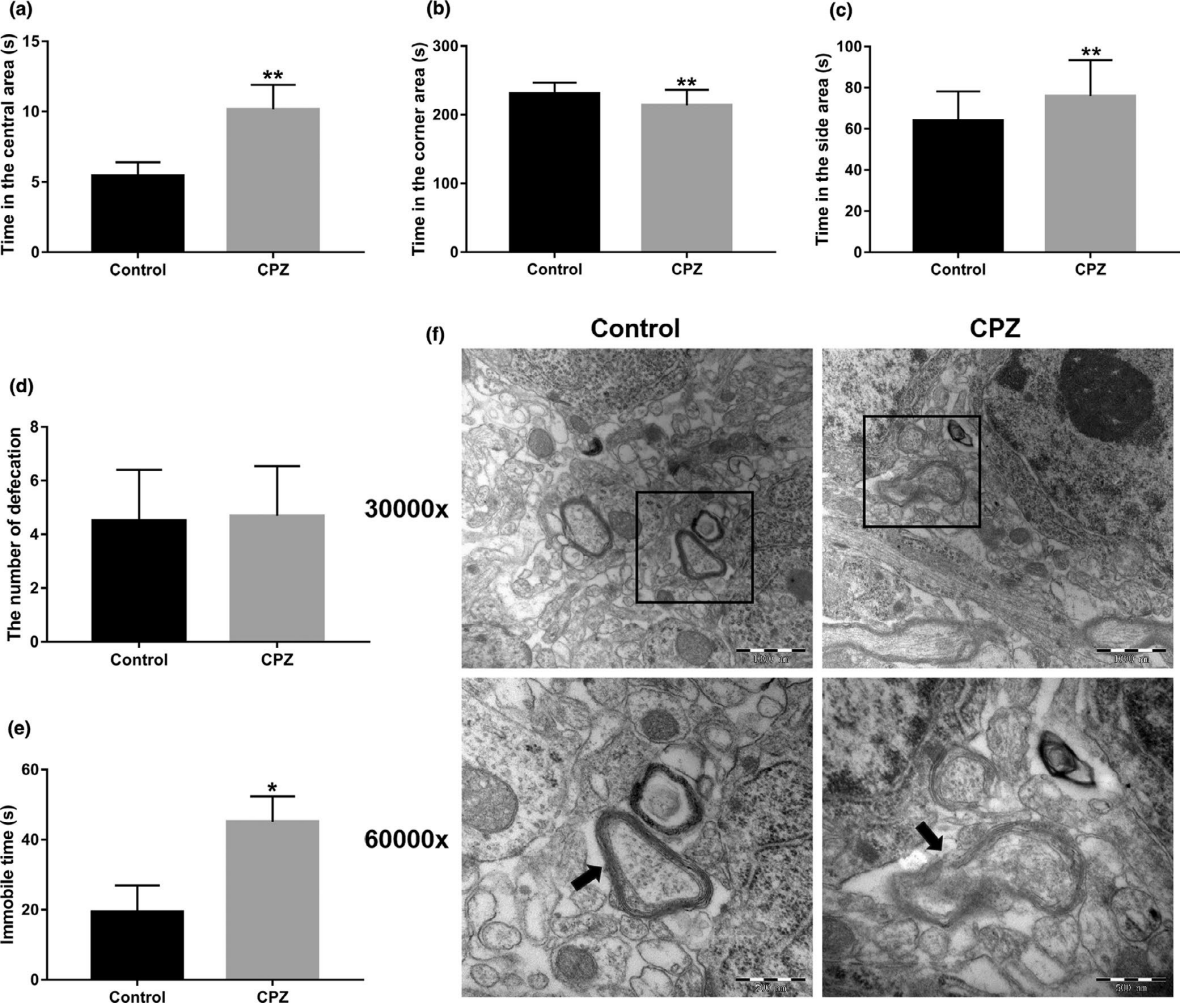
Effect of CPZ on the behavior of mice and the ultrastructure of hippocampal myelination. (a) Time in the central area in the OFT of CPZ‐induced demyelinated mice. (b) Time in the corner area in the OFT of CPZ‐induced demyelinated mice. (c) Time in the side area in the OFT of CPZ‐induced demyelinated mice. (d) Number of defecations in the OFT of CPZ‐induced demyelinated mice. (e) Immobile time in the TST of CPZ‐induced demyelinated mice. (f) Ultrastructure of hippocampal myelin sheaths of CPZ‐induced demyelinated mice. 30,000 x magnification, Scale bar = 1,000 nm. 60,000 x magnification, scale bar = 500 nm, arrow indicates the location of the myelin sheath. Data are presented as mean ± *SEM*, *p < .05, **p < .01. OFT: open field test; TST: tail suspension test

In the OFT, mice of the CPZ group spent more time in the central area (*p* < .01; Figure 1a) and less in the corner and side area than control mice (*p* < .01; Figure 1b,c). No significant difference in the number of defecations was found (Figure 1d). In the TST, the immobile time of the CPZ group was significantly increased compared with control mice (*p* < .05; Figure 1e). TEM data showed that the hippocampal myelin lamina of the control group was uniform in thickness and closely arranged, and the organelles inside the axon were normal in shape and size. In contrast, a large number of pathological myelin sheaths occurred in the CPZ group, which was manifested by myelin edema, increased space between myelin sheaths and axons, and vacuoles. The plate was separated and fractured, the myelin sheaths were disintegrated, and the myelin sheath structure disappeared (Figure 1f).

### 3.2 Effect of CPZ on the expression of hippocampal myelin‐related protein in mice

**Figure 2:**
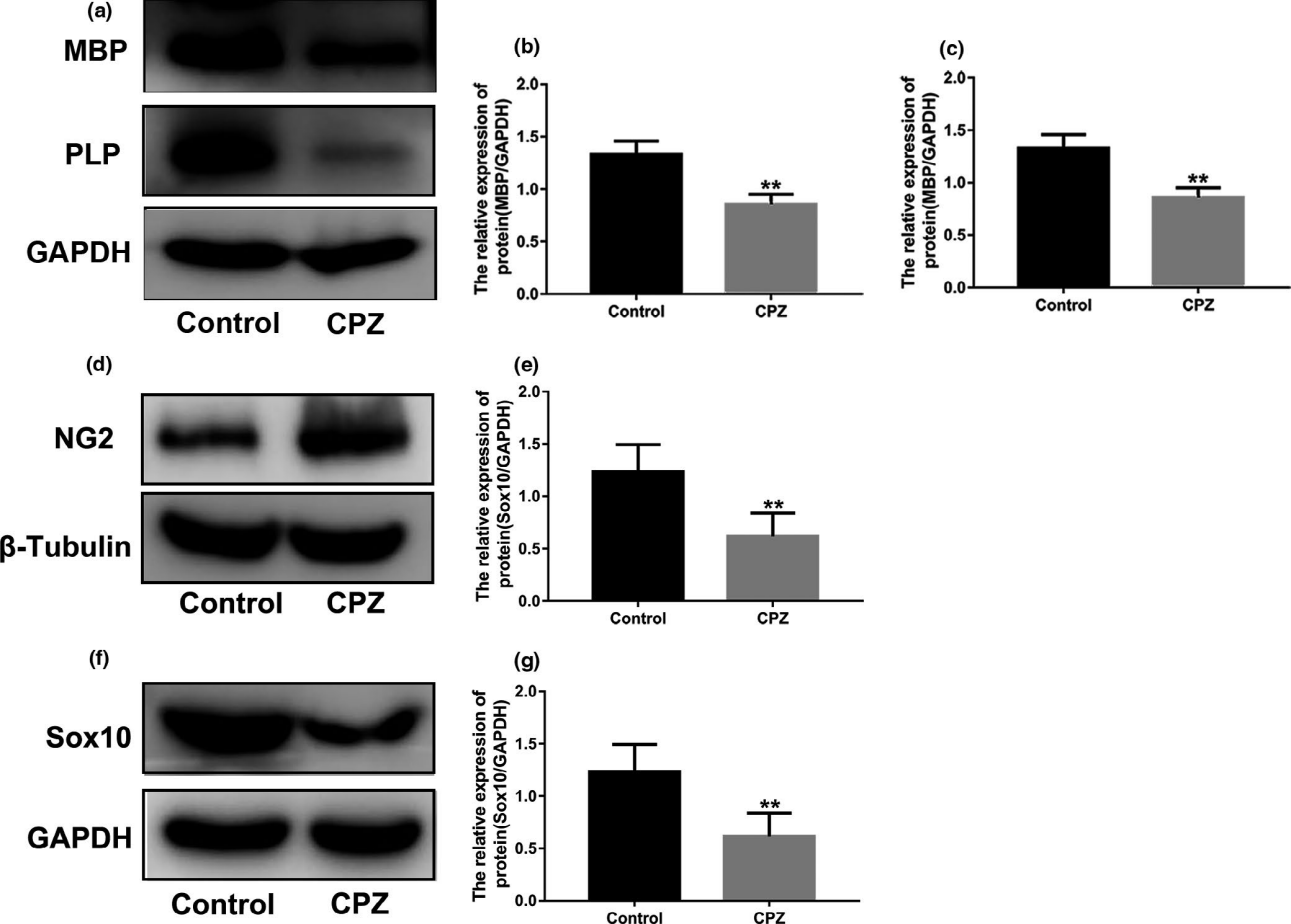
Effect of CPZ on the expression of hippocampal myelin‐related protein in mice. (a) Expression of MBP and PLP in CPZ‐induced demyelinated mice. (b,c) Immunoblot analysis of MBP and PLP expression in CPZ‐induced demyelinated mice. (d) Expression of NG2 in CPZ‐induced demyelinated mice. (e) Immunoblot analysis of NG2 expression in CPZ‐induced demyelinated mice. (f) Expression of Sox10 in CPZ‐induced demyelinated mice. (g) Immunoblot analysis of Sox10 expression in CPZ‐induced demyelinated mice. Data are presented as mean ± *SEM*, *p < .05, **p < .01

MBP and myelin PLP are markers of mature OLs. The expression of hippocampal MBP and PLP in the CPZ group was lower than in the control group (*p* < .01; Figure 2a–c), whereas the expression of the OPC marker NG2 was higher than in the control group (*p* < .01; Figure 2d,e). After demyelination, the expression of Sox10 was lower in the CPZ group than in the control group (*p* < .01; Figure 2f,g).

### 3.3 Identification of Sox10 overexpression through AAV infection

**Figure 3:**
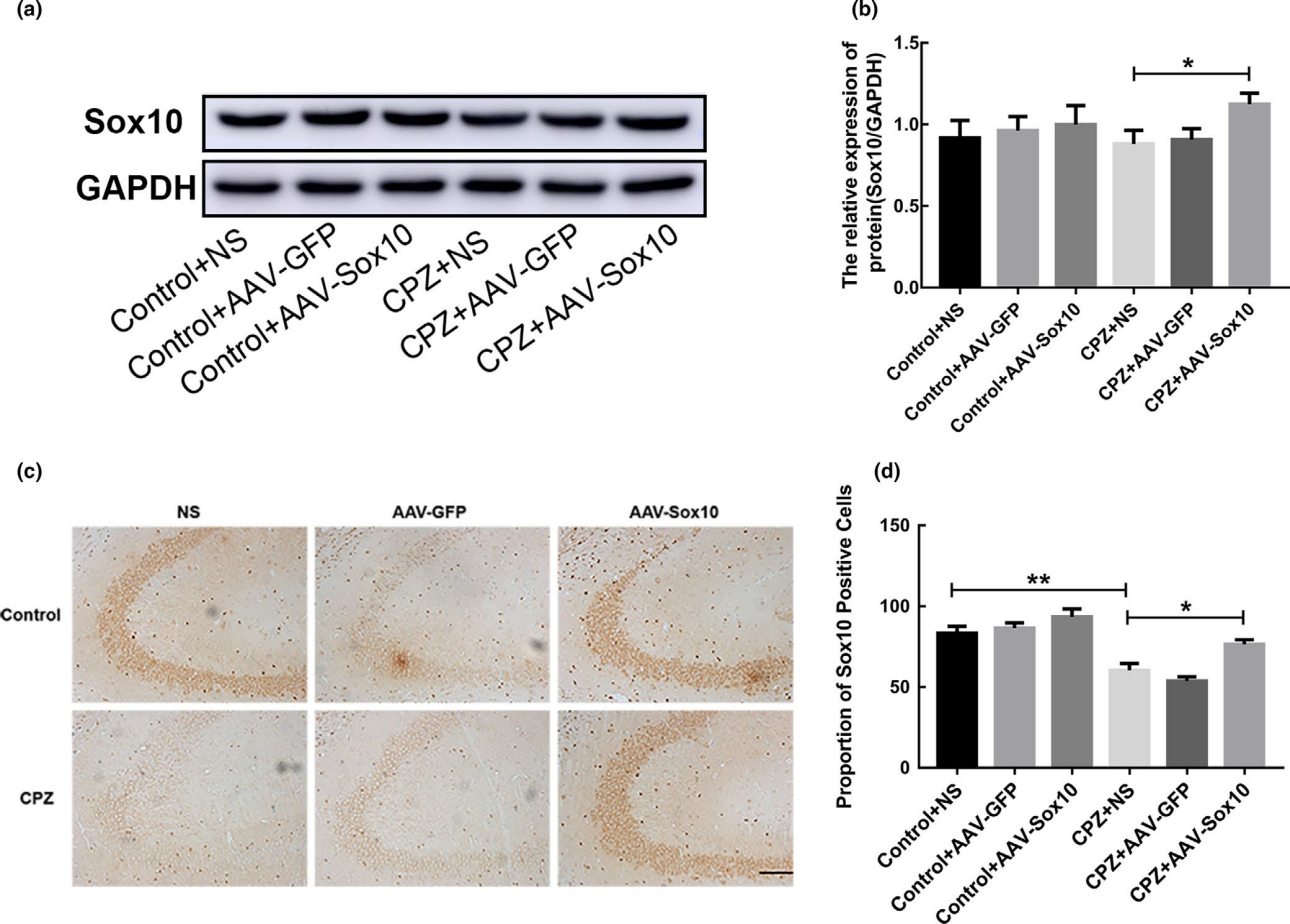
Identification of Sox10 overexpression through AAV infection. (a) Expression of Sox10 in mice. (b) Immunoblot analysis of Sox10 expression. (c) Expression of Sox10 detected by immunohistochemistry. Scale bar = 100 μm. (d) Statistical analysis of the number of Sox10‐positive cells. Data are presented as mean ± *SEM*, *p < .05, **p < .01

Stereotactic intracranial injection of viral vectors is an effective technique for direct delivery of genetic material to specific cell populations in the CNS of mice [43]. In the present study, AAV with the best in vivo infection efficiency [44] was selected for Sox10 delivery. After the injection of AAV‐Sox10, the expression of Sox10 increased in the hippocampus of mice (*p* < .01; Figure 3a–d).

### 3.4 Overexpression of Sox10 improves the behavior and ultrastructure of hippocampal myelin in CPZ mice

**Figure 4:**
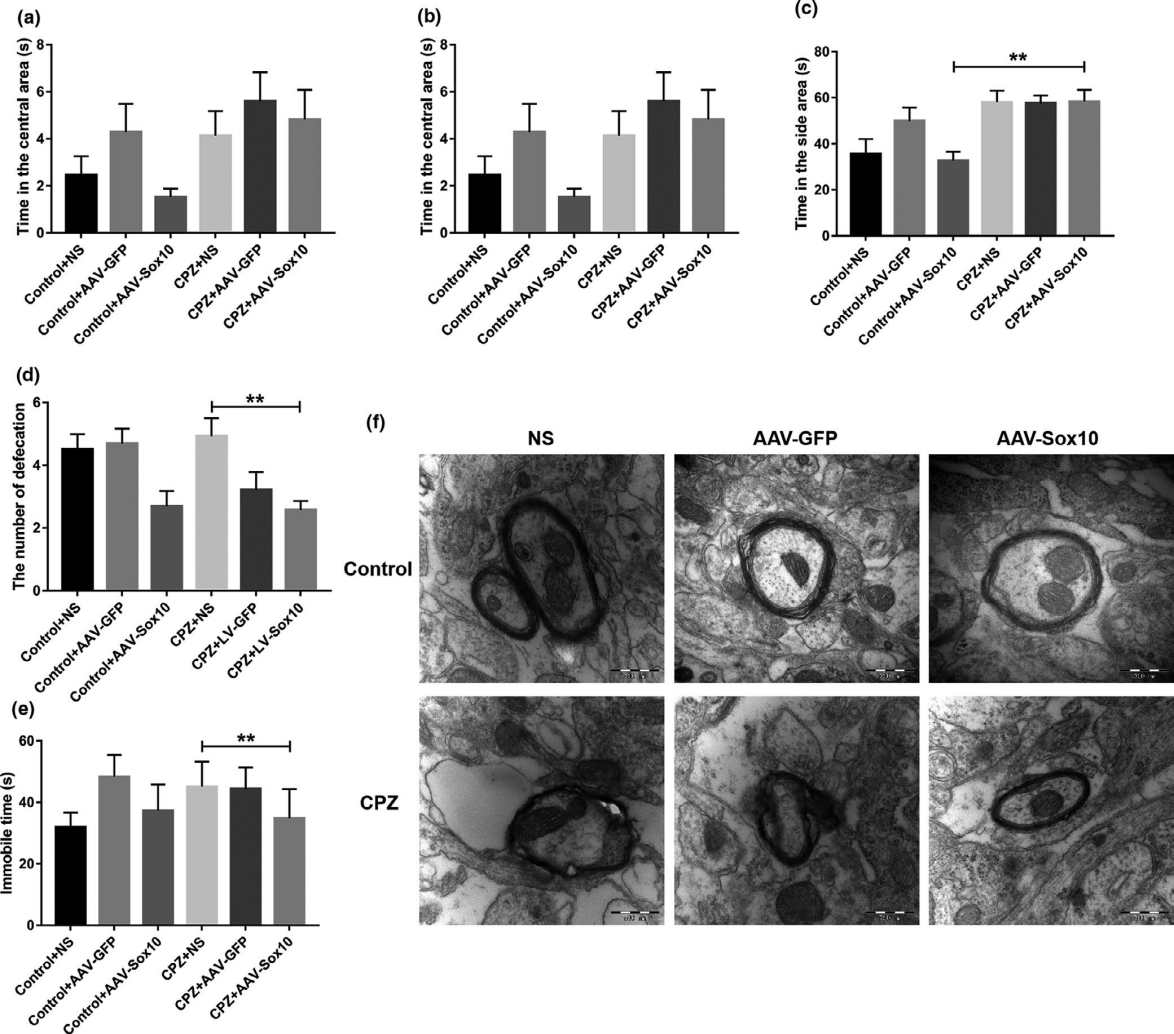
Overexpression of Sox10 improves the behavior and ultrastructure of hippocampal myelin in CPZ‐induced demyelinated mice. (a) Time in the central area in the OFT. (b) Time in the corner area in the OFT. (c) Time in the side area in the OFT. (d) Number of defecations in the OFT. (e) Immobile time in the TST. (f) Ultrastructure of hippocampal myelin sheaths. Scale bar = 500 nm. Data are presented as mean ± *SEM*, *p < .05, **p < .01. OFT: open field test; TST: tail suspension test

No significant differences in the time of each area of CPZ mice were found among the different groups (Figure 4a–c). After overexpression of Sox10 in demyelinated mice, the number of defecations in the OFT (*p* < .01; Figure 4d) and the immobile time in the TST (*p* < .01; Figure 4e) significantly improved. In the control group, the myelin sheath of the hippocampus was normal, the myelin axons were tightly connected, and no abnormalities were observed in the axons and their organelles. Pathological changes in the ultrastructure of myelin were observed in the demyelinated mice. No significant difference in myelin ultrastructure was found between the normal control group and the Sox10‐overexpression group (Figure 4f).

### 3.5 Overexpression of Sox10 promotes the expression of myelin sheath‐related protein in CPZ mice

**Figure 5:**
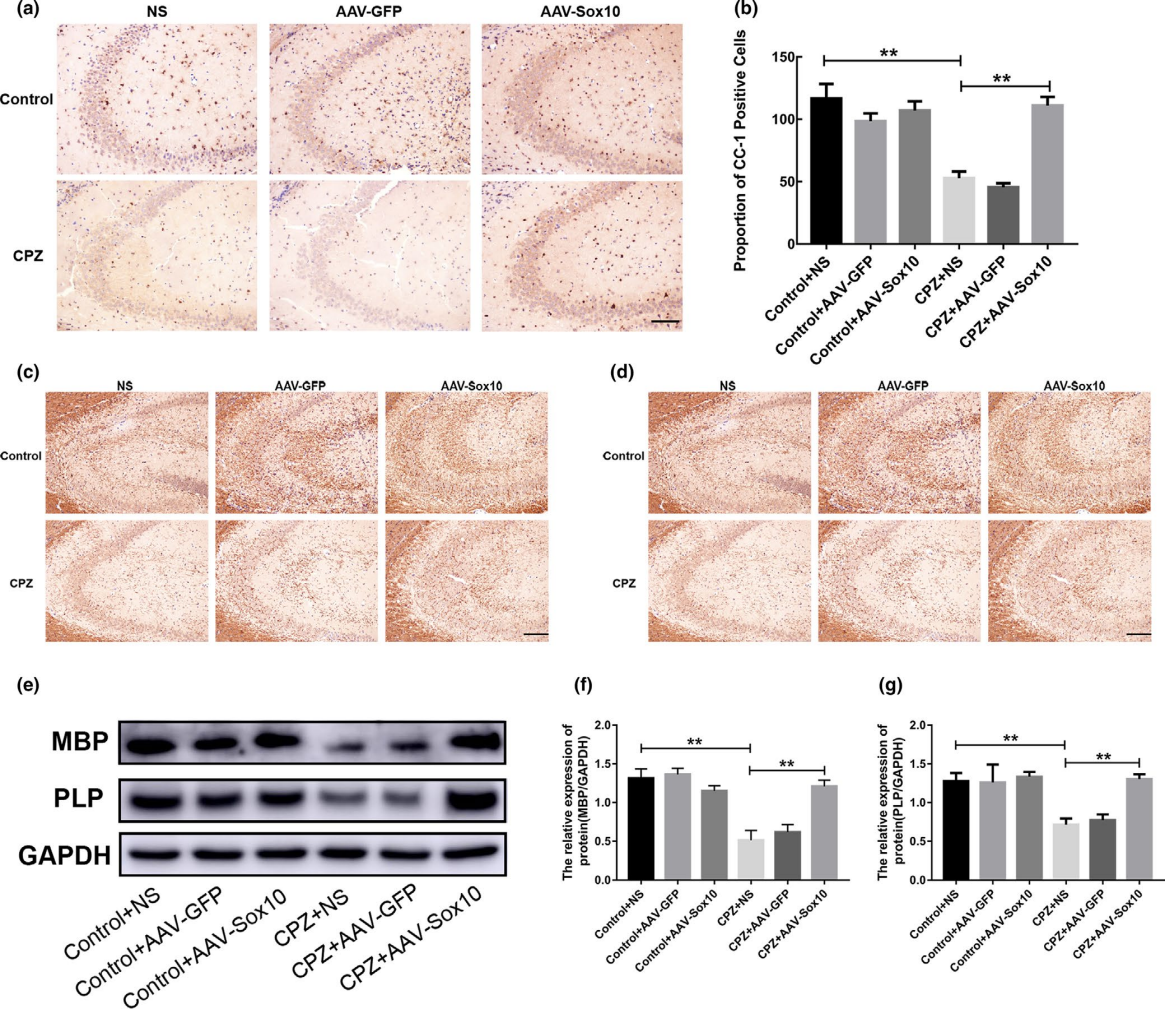
Overexpression of Sox10 promotes the expression of myelin sheath‐related protein in CPZ‐induced demyelinated mice. (a) Immunohistochemical detection of CC‐1 expression. Scale bar = 100 μm. (b) Statistical analysis of the number of CC‐1‐positive cells. (c) Immunohistochemical detection of MBP expression. Scale bar = 100 μm. (d) Immunohistochemical detection of PLP expression. Scale bar = 100 μm. (e) MBP and PLP expression detected by Western blot. (f) Immunoblot analysis of MBP expression. (g) Immunoblot analysis of PLP expression. Data are presented as mean ± *SEM*, *p < .05, **p < .01

No significant difference in the expression of myelin marker protein was found in the control group after the different treatments. Two weeks after the removal of CPZ, the expression of the myelin marker proteins in the CPZ group was still lower than that in the control group. The CPZ group had a large number of adenomatous polyposis coli (APC, also known as CC‐1)‐positive cells after overexpressing Sox10 (*p* < .01; Figure 5a,b). The expression of MBP and PLP increased after overexpression of Sox10 (*p* < .01; Figure 5c–g).

### 3.6 Overexpression of Sox10 promotes OPC marker protein NG2 expression in CPZ mice

**Figure 6:**
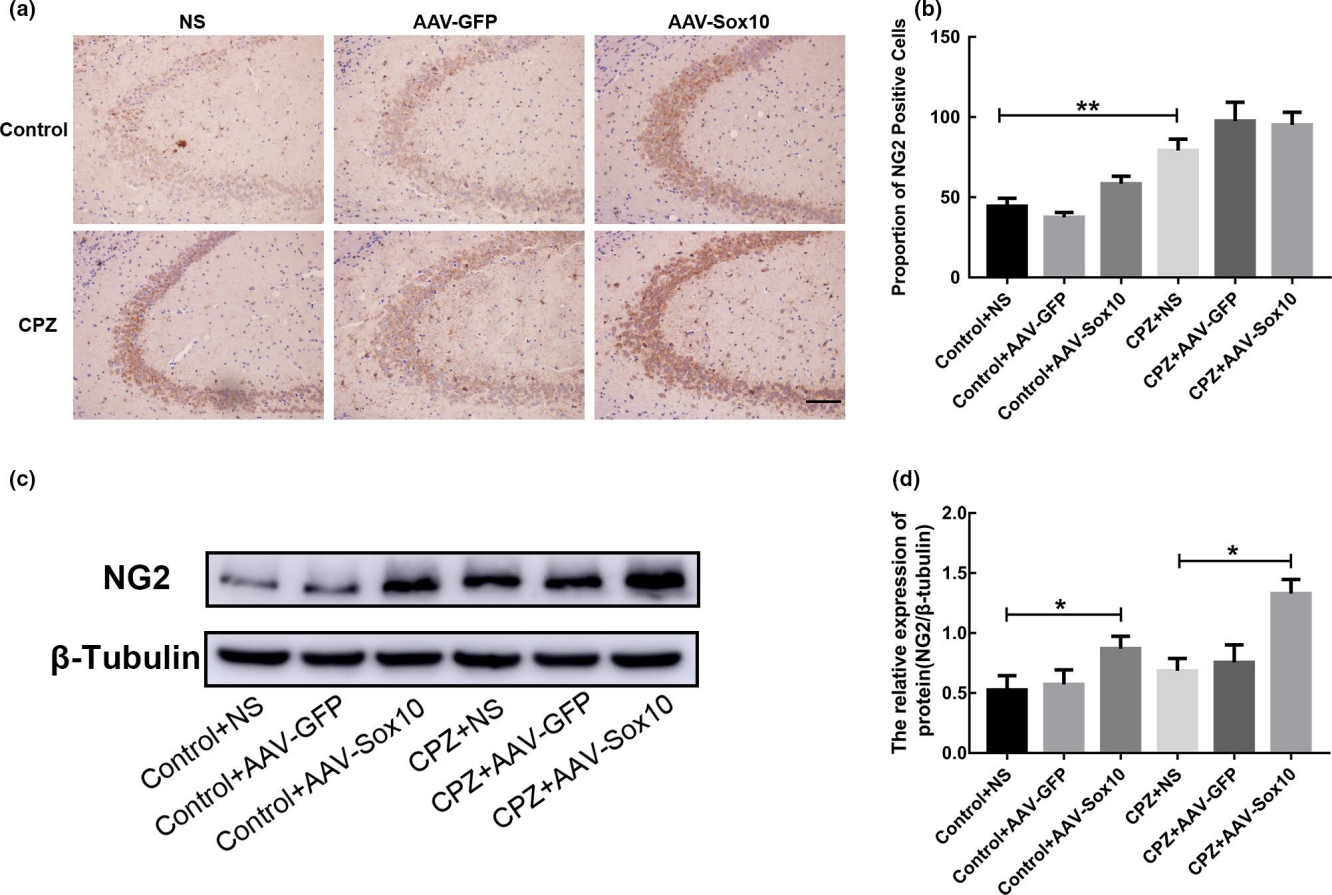
Overexpression of Sox10 increases expression of the OPC marker protein NG2 in CPZ‐induced demyelinated mice. (a) Immunohistochemical detection of NG2 expression. Scale bar = 100 μm. (b) Statistical analysis of the number of NG2‐positive cells. (c) Expression of NG2 detected by Western blot. (d) Immunoblot analysis of NG2 expression. Data are presented as mean ± *SEM*, *p < .05, **p < .01

Without Sox10 overexpression, NG2 expression was higher in the CPZ group (*p* < .01; Figure 6a,b). Demyelinated and normal mice showed increased NG2 expression after enhanced Sox10 expression (*p* < .05; Figure 6c,d).

## 4 DISCUSSION

Schizophrenia is a common psychiatric disease whose etiology has not yet been fully revealed. It often occurs in young adults, impairing thinking, perception, emotion, and behavior, and can be incompatible with mental activities and the environment (Cui et al., 2019). A variety of genes associated with schizophrenia are highly expressed in the hippocampus (Kaefer et al., 2019) which also reflects the importance of the hippocampus for studying schizophrenia. The hippocampus is highly susceptible to injury by many injury factors (Kutlu & Gould, 2016). Myelin formation begins before birth and continues after birth until adolescence. Schizophrenia is more common in adolescence (Gur et al., 2019). Therefore, the molecular mechanism of pathological changes in myelination of the hippocampus is important for exploring the pathogenesis of schizophrenia. In demyelinated patients, Sox10 expression is decreased and OPC differentiation is blocked (Zeis, Howell, Reynolds, & Schaeren‐Wiemers, 2018). Our study results showed that the expression of Sox10 significantly decreased in the CPZ mice. To reveal the regulatory role of Sox10 in hippocampal myelin regeneration, we injected Sox10‐overexpressing AAV into the hippocampus region of mice via a stereotaxic apparatus and confirmed overexpression of Sox10 in the hippocampus through immunohistochemistry and Western blots. We further studied the effect of Sox10 overexpression on hippocampal remyelination in CPZ mice. In this study, we aimed to explore a way to promote myelin regeneration.

Normal mice feel fear of the unfamiliar environment and stay only for a short time in the open central area in the OFT. Our study showed that the time CPZ mice spent in the central area was significantly increased compared with control mice, indicating that CPZ mice displayed abnormal anxiety. In CPZ mice, demyelination reduced the novel anxiety reaction (Tomas‐Roig, Torrente, Cabre, Vilella, & Colomina, 2019), which led to increased time spent in the central region. The depression status of CPZ mice was significantly worse than that of the normal mice. This result is consistent with the findings of Pusic et al (Pusic, Mitchell, Kunkler, Klauer, & Kraig, 2015), confirming the abnormal behavioral changes in CPZ‐induced demyelinated mice. However, the molecular mechanism of behavioral changes is not clear. This is the first study available on the effects of Sox10 on behavior in the CPZ mouse model. Our data showed that overexpression of Sox10 improved the degree of stress and depression in the CPZ‐induced demyelinated mice. A study by Zendedel (Zendedel, Beyer, & Kipp, 2013) reported the relationship between remyelination and axonal injury and found that the animal's motor function began to recover when CPZ was removed after CPZ was fed for five weeks but the animal's motor performance was still impaired even six months after CPZ withdrawal. We overexpressed Sox10 in CPZ mice. After two weeks, the behavioral indexes of these mice were not different from those of the control group. This result indicates that Sox10 can improve the behavioral function of demyelinated mice.

The results of TEM showed a large number of diseased myelin sheaths in the hippocampus of CPZ mice and different degrees of edema, pathological changes such as loosening, lamellar separation, and axonal separation of myelin, and even complete loss of the myelin structure. Similar pathological changes have been reported previously (Aryanpour et al., 2017). Changes in myelin ultrastructure confirmed that hippocampal myelin was seriously damaged. The demyelination model was successfully established and confirmed via the observation of behavior and the ultrastructure of the hippocampal myelin sheath. Overexpression of Sox10 restored the damaged myelin ultrastructure to the normal structure with no significant difference from the control group. Therefore, Sox10 overexpression can promote restoration of the myelin sheath structure.

We further examined the expression of myelin marker proteins. CC‐1, MBP, and PLP are markers of mature OLs, in which CC‐1 is expressed on the cell body, and OLs can be counted by staining with morphology. MBP and PLP are myelin‐forming proteins secreted by OLs, which are morphologically filamentous because they encapsulate axons. In demyelinating lesions, their expression can represent the degree of myelin damage (Burda, Radulovic, Yoon, & Scarisbrick, 2013). Immunohistochemical and Western blot results showed that the expression levels of CC‐1, MBP, and PLP were significantly decreased in CPZ mice. These results confirmed the successful establishment of the mouse hippocampal demyelination model. Remyelination after demyelination can theoretically be achieved by enhancing endogenous remyelination or by transplanting exogenous myelin‐forming cells. Previous studies on promoting endogenous remyelination focused on how to enhance the survival of OPCs and stimulate their proliferation, migration, and differentiation into myelin cells (Grade, Bernardino, & Malva, 2013; Kastriti & Adameyko, 2017). In our study, OPCs were found around the demyelinating lesions in the CNS. However, they all remained at a static stage, unable to proliferate and differentiate into mature OLs, and thus unable to form a new myelin sheath to wrap the exposed axons. NG2, a marker of OPCs, is used to reflect the presence of OPCs. Our study showed that the expression of NG2 increased in CPZ mice. Furthermore, the number of OPCs increased after demyelination, which may be the repair response of the body to pathological demyelination. However, despite the increase in OPCs, the expression of MBP and other myelin markers still decreased. This indicates that OPCs after demyelination have not effectively differentiated into mature oligodendrocytes. OPCs cannot differentiate and repair the myelin sheath, which is considered as the major obstacle to successful repair of demyelinating lesions (Dulamea, 2017). In this study, after overexpression of Sox10, the expression levels of myelin maturation markers CC‐1, MBP, and PLP significantly increased. Additionally, the expression of NG2 increased in demyelinated mice. This result indicates that Sox10 not only promotes the differentiation of OPCs but also promotes the production of OPCs through other ways, which can supplement the consumption of OPCs caused by differentiation to solve the problem of insufficient remyelination. A previous study has shown that myelin sheaths can be fully restored after the removal of CPZ for 3–5 weeks (Wergeland, Torkildsen, Myhr, Mork, & Bo, 2012). In our study, Sox10 was overexpressed immediately after the demyelinating model was established and this promoted OLs to secrete myelin protein again after two weeks at the same level as the control group. This result indicated that after the removal of CPZ, Sox10 significantly enhanced the expression of myelin protein to support self‐repair of the myelin sheaths.

It should be noted that our study has examined only the myelin marker proteins CC‐1, MBP, and PLP and the OPC marker NG2 to investigate the effect of Sox10 on hippocampal remyelination. We did not explore Sox10 signaling pathways. Future studies will be needed to address this.

## 5 CONCLUSION

In summary, in the CPZ‐induced acute demyelinating mouse model, the expression of Sox10 and the expression of myelin marker proteins CC‐1, MBP, and PLP were downregulated. Overexpression of Sox10 significantly improved the behavior, myelin morphology, myelin marker protein expression, and the number of OPCs in CPZ‐induced demyelinated mice. Therefore, Sox10 plays an important role in regulating the regeneration of hippocampal myelin sheaths. It also provides a new therapeutic target for the treatment of myelin sheath injury in schizophrenia.

## CONFLICT OF INTEREST

The authors declare no conflicts of interest.

## AUTHORS CONTRIBUTION

YS performed the experiments. JD performed the data analyses and drafted the manuscript. QXH assisted with the experiments. QRM, QL, CZ, and HWL collected and interpreted the data. JL conceived the study. All authors read and approved of the final manuscript.

## Data Availability

The data that support the findings of this study are available from the corresponding author upon reasonable request.
